# Telomere length and telomerase activity in T cells are biomarkers of high‐performing centenarians

**DOI:** 10.1111/acel.12859

**Published:** 2018-11-28

**Authors:** Enzo Tedone, Ejun Huang, Ryan O’Hara, Kimberly Batten, Andrew T. Ludlow, Tsung‐Po Lai, Beatrice Arosio, Daniela Mari, Woodring E. Wright, Jerry W. Shay

**Affiliations:** ^1^ Department of Cell Biology UT Southwestern Medical Center Dallas Texas; ^2^ Geriatric Unit, Department of Medical Sciences and Community Health University of Milan Milan Italy; ^3^ Fondazione Ca' Granda, IRCCS Ospedale Maggiore Policlinico Milan Italy

**Keywords:** centenarians, healthy aging, longevity, stimulated T cells, telomerase activity, telomeres

## Abstract

It is generally recognized that the function of the immune system declines with increased age and one of the major immune changes is impaired T‐cell responses upon antigen presentation/stimulation. Some “high‐performing” centenarians (100+ years old) are remarkably successful in escaping, or largely postponing, major age‐related diseases. However, the majority of centenarians (“low‐performing”) have experienced these pathologies and are forced to reside in long‐term nursing facilities. Previous studies have pooled all centenarians examining heterogeneous populations of resting/unstimulated peripheral blood mononuclear cells (PBMCs). T cells represent around 60% of PBMC and are in a quiescence state when unstimulated. However, upon stimulation, T cells rapidly divide and exhibit dramatic changes in gene expression. We have compared stimulated T‐cell responses and identified a set of transcripts expressed in vitro that are dramatically different in high‐ vs. low‐performing centenarians. We have also identified several other measurements that are different between high‐ and low‐performing centenarians: (a) The amount of proliferation following in vitro stimulation is dramatically greater in high‐performing centenarians compared to 67‐ to 83‐year‐old controls and low‐performing centenarians; (b) telomere length is greater in the high‐performing centenarians; and (c) telomerase activity following stimulation is greater in the high‐performing centenarians. In addition, we have validated a number of genes whose expression is directly related to telomere length and these are potential fundamental biomarkers of aging that may influence the risk and progression of multiple aging conditions.

## 1 INTRODUCTION

There have been longitudinal human studies demonstrating specific changes in immune responses that correlate with many causes of increased morbidity and mortality with increased age (Wikby et al., 2006). For example, it is already known that many older individuals (>65 years old) have impaired responses to pathogens such as influenza virus (Murphy, Xu, & Kochanek, 2013). Thus, it is important to better understand the underlying biological mechanisms that contribute to age‐related impairment of immune functions in order to develop appropriate preventative and therapeutic strategies to meet the medical and health demands of an increasing elderly population worldwide.

Some centenarians (individuals who are at least 100 years old) reach what is currently the extreme limit of human life by escaping, or largely postponing, the major age‐related diseases and retaining physical independence for an extraordinary long period. For these reasons, they can be considered a paradigm for the study of healthy aging. Previous studies of long‐lived families have been carried out from several perspectives, such as genetics (Beekman et al., 2013), epigenetics (Gentilini et al., 2013), and transcriptomics (Passtoors et al., 2012), leading to the identification of characteristic profiles of both longevity and healthy aging. However, these investigations have focused on the behavior of resting immune cells (e.g., peripheral blood mononuclear cells, PBMCs). Peripheral blood mononuclear cells are a heterogeneous cell population mainly consisting of T cells, a major component of human immune responses. T cells remain in a resting or quiescent state when unstimulated, showing little or no proliferation activity. In contrast, upon antigen‐specific activation, T cells rapidly divide and exhibit dramatic changes in gene expression (over 7,000 genes are differentially expressed in resting vs. stimulated T cells) (Zhao, Fung‐Leung, Bittner, Ngo, & Liu, 2014).

Activated T cells initiate immune responses such as discriminating between healthy and abnormal (e.g., infected or cancerous) cells in the body and thus represent a model to study the physiological function of the adaptive immune system (including inflammatory signaling). Comparing stimulated T‐cell responses in different age groups including centenarians is an understudied area of research and might thus provide new insights about the mechanisms driving the age‐related impairment of the immune system as well as the mechanisms responsible for at least a subset of centenarians’ extremely healthy aging.

With the goal of identifying genes and pathways potentially involved in the process of healthy aging and longevity, this series of pilot studies involving 114 individuals was designed to characterize and compare the stimulation‐induced responses in T cells from 19 young (23–39 years old), 48 middle‐aged (50–66 years old), 26 old (67–83 years old), and 21 very old (centenarians: 100+ years old) individuals. We used whole genome RNA‐sequencing, telomerase activity, and telomere length assays to compare the function of stimulated T cells from a subset of the study groups listed above. Based on these preliminary investigations, we are now able to separate low‐performing from high‐performing centenarians and potentially disentangle age‐associated pathology from a set of promising underlying biochemical biomarkers of healthy aging.

## 2 RESULTS

### 2.1 Stimulated T cells from centenarians show robust telomerase activity and replicative potential

Telomeres are tandem repeats that cap the end of linear chromosomes to protect them from degradation and to prevent chromosome fusions (Shay, 2016). In normal human proliferating cells, telomeres get progressively shorter with each cell division, leading eventually to DNA damage responses, replicative senescence, or apoptosis (Shay, 2016). One consequence of proliferation is that telomere length declines with age and is considered a marker of biological (not chronological) age (Epel et al., 2009) that correlates with various age‐related pathologies including cancer (Shay, 2016), dementia (Tedone et al., 2015), and cardiovascular diseases (Epel et al., 2009). However, some cell types such as proliferating stem‐like cells and antigen‐stimulated T cells express regulated telomerase activity, a ribonucleoprotein enzyme complex whose specialized reverse transcriptase action performs de novo addition of telomeric TTAGGG repeats at the end of telomeres, thus slowing down the rate of telomere attrition that occurs during cell division (Shay, 2016).

**Figure 1:**
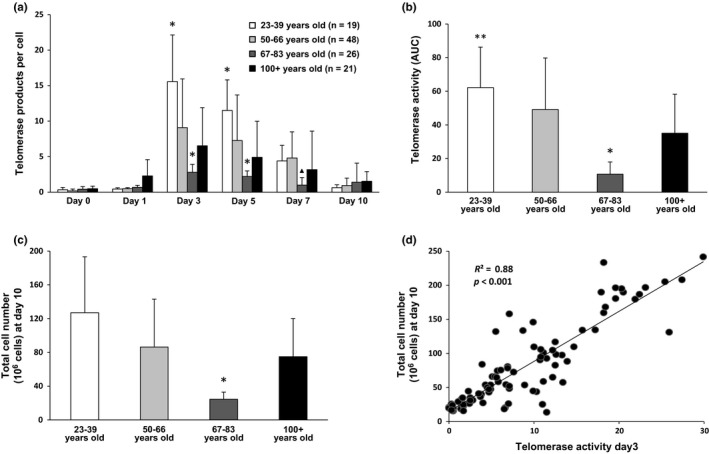
Telomerase activity and proliferation capacity in stimulated peripheral blood mononuclear cell (PBMC). (a) Telomerase activity measured by ddTRAP on stimulated PBMC at Days 0, 1, 3, 5, 7, and 10 after stimulation with anti‐CD3/anti‐CD28 Dynabeads. *p < 0.05 vs. each of the other groups. ^▲^ *p* < 0.05 vs. both 23–39 years old and 50–66 years old (b) Total telomerase activity (area under the curve, AUC) over a 10‐day period of stimulation. *p < 0.05 vs. each of the other groups. **p < 0.05 vs. centenarians. (c) Total cell number after 10 days of stimulation (for all samples, two million PBMCs were stimulated at day 0). *p < 0.05 vs. each of the other groups. (d) Correlation between telomerase activity at Day 3 after stimulation and total cell number at Day 10

Our previous study on unstimulated leukocytes isolated from peripheral blood showed that a subset of centenarians underwent a slower rate of telomere shortening compared with the normal population (Tedone et al., 2014). We hypothesized that in some centenarians, T cells have increased telomerase activity leading, in turn, to better telomere length maintenance mechanisms. In the present studies, we employed a droplet digital PCR assay (the ddTRAP assay) (Ludlow et al., 2014) to measure telomerase activity. This assay provides an absolute quantification of telomerase activity even at the single cell level. Unstimulated T cells (day 0) showed extremely low telomerase activity, and no significant differences were found between the study groups (Figure 1a). Consistent with a previous study (Huang et al., 2017), upon T‐cell stimulation, telomerase activity was transiently up‐regulated, generally peaking 3–5 days after stimulation and then slowly declining (Figure 1a). We calculated the magnitude of telomerase activity over a 10‐day period following stimulation by measuring, for each study group, the area under the curve (AUC) of Figure 1a,b. Telomerase activity in stimulated T cells from our pool of 21 centenarians was significantly higher compared to 67‐ to 83‐year‐old individuals and was comparable to that of 50‐ to 66‐year‐old individuals (Figure 1a,b).

In addition, the total number of T cells produced was greater for centenarians compared with those 67–83 years old (Figure 1c). Since telomerase is believed to increase the cellular replicative lifespan through telomere maintenance, we investigated whether telomerase activity correlated with the number of cell divisions. We found a significant correlation between T‐cell telomerase activity at Day 3 of stimulation (peak) and the maximal number of cells (a proxy for the rate of cell division) over a 10‐day period (Figure 1d). This suggests that telomerase activity in stimulated T cells may be a biomarker of the magnitude of stimulation‐induced responses. The combined results show that in our study population, stimulated T cells from centenarians express higher levels of telomerase activity and proliferate better than T cells from younger unrelated old individuals (67–83 years old).

### 2.2 Some centenarians have global genome expression profiles more similar to that of young adults

**Figure 2:**
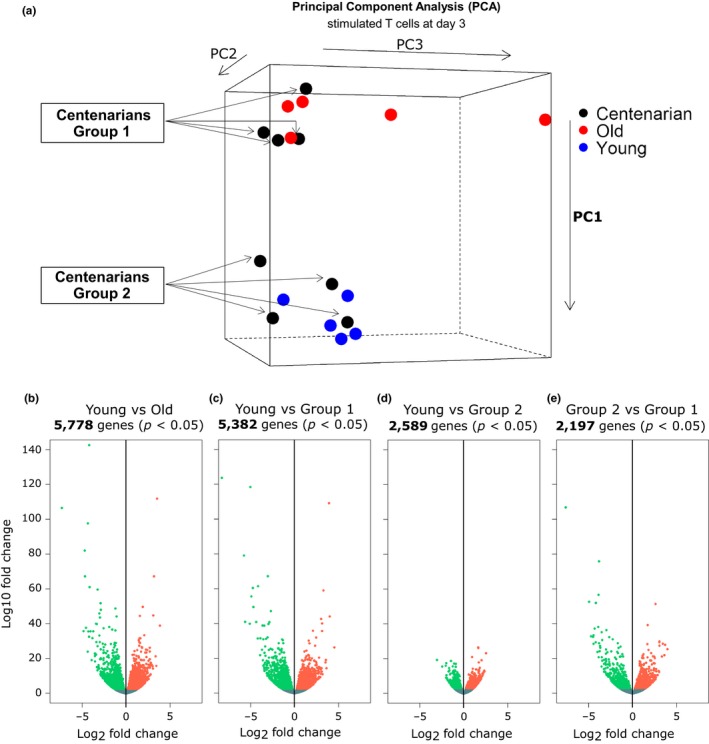
Global gene expression profile comparisons between young, old (67–83 years old), and centenarians. (a) Principal component analysis (PCA) representing young, old (67–83 years old), and the two subsets of centenarians (Group 1 and Group 2). (b–d) Volcano plots of genes differentially expressed (*p* < 0.05) between young vs. old, and young vs. the two subsets of centenarians (Group 1 and Group 2). (e) Volcano plots of genes differentially expressed (*p* < 0.05) between the two subsets of centenarians (Group 1 vs. Group 2)

To identify genes potentially associated with the centenarians’ T‐cell phenotype, we compared in a subset of our study population the global gene expression profiles of stimulated T cells by RNA‐sequencing. Since most of the centenarians were females, we only analyzed women in order to avoid confounding factors that depend on gender. In our initial exploratory RNA‐seq analysis, we investigated whether in stimulated T cells, there existed characteristic expression profiles defining young (23–39 years old), old (67–83 years old), and centenarians. We used both principal component analysis (PCA) and unsupervised hierarchical clustering (HCL) to visualize the data. In both PCA and HCL, we noticed the same division among sample (Figure 2a, Supporting Information Figure S1), and the three‐dimensional representation (PCA) was chosen to illustrate this finding that ultimately guided our subsequent DESeq2 analysis. As might be expected, both young and old individuals exhibited very different gene expression profiles (Figure 2a). Interestingly, the centenarian group was heterogeneous, with some centenarians “Group 1” clustering with the old and some centenarians “Group 2” clustering with the young individuals (Figure 2a). When compared to young healthy subjects, both old (67–83 years old) and centenarians Group 1 displayed more than 5,000 genes differentially expressed (5,778 and 5,382 genes, respectively, *p* ≤ 0.05) (Figure 2b,c). However, centenarians Group 2 had fewer genes differentially expressed (2,589 genes, *p* ≤ 0.05) and with less dramatic fold change differences (Figure 2d). Consistent with the PCA analysis, stimulated T cells from Group 2 centenarians also had 2,197 genes whose expression level was significantly different compared to Group 1 centenarians (Figure 2e).

After completing these initial transcriptomic analyses, we checked the health status (medical records) and both cognitive and physical performances, respectively, measured by using the Mini‐Mental State Examination (MMSE) test and the Lawton Instrumental Activities of Daily Living (IADL) test (Folstein, Folstein, & McHugh, 1975; Lawton & Brody, 1969). Interestingly, Group 2 centenarians (that clustered with the young healthy group) had significantly better cognitive and physical performances and were also affected by a significantly lower number of age‐related diseases compared with Group 1 centenarians (that clustered with the old‐aged group) (Table 1). Together, we interpret these results to suggest that combining all centenarians into one group is likely to give heterogeneous results.

**Table 1:** Health status assessment of the individuals selected for global genome expression profile analysis by RNA‐sequencing. Disease count was determined by evaluating the presence of the following diseases: Acute myocardial infarction, stroke, angina, hypertension, COPD, dementia, depression, diabetes, thyroid dysfunction, arthrosis, chronic liver diseases, and chronic kidney diseases

Health status assessment	Centenarians group 1 (*n* = 4)	Centenarians group 2 (*n* = 4)	Old subjects (*n* = 5)	Young subjects (*n* = 5)	*p* Value
Age, years, mean ± *SD*	103.8 ± 2.5	103.5 ± 3.1	75.0 ± 4.2^a^	24.5 ± 2.1^a^	<0.001
Gender, female, %	100	100	100	100	NA
Smokers, %	0	0	0	0	NA
Body mass index (BMI), mean ± *SD*	22.7 ± 2.5	25.1 ± 3.0	26.0 ± 4.3	24.5 ± 5.3	0.720
Cognitive performance, MMSE score (0–30), mean ± *SD*	14.2 ± 13.3^a^	28.0 ± 1.4	30.0 ± 0.0	30.0 ± 0.0	0.001
Physical performance, IADL score (0–8), mean ± *SD*	1.8 ± 1.0^a^	6.8 ± 1.5	8.0 ± 0.0	8.0 ± 0.0	<0.001
Disease count per individual, mean ± *SD*	6.0 ± 0.8^a^	2.5 ± 0.6^b^	1.0 ± 0.7	0.0 ± 0.0	<0.001

a *p* < 0.05 vs. each of the other groups.

b *p* < 0.05 vs. young subjects.

### 2.3 Healthier centenarians clustering with the young also have significantly longer telomeres compared to their centenarian peers

Telomere length is believed to be a marker of biological age and exposure to various age‐related diseases (Epel et al., 2009; Shay, 2016). Since Group 2 centenarians were by far healthier than Group 1 centenarians (Table 1), we next investigated whether they also had longer T‐cell telomeres. We measured both the average telomere length and the length of the shortest 20% telomeres using a recently developed highly sensitive assay (TeSLA, Telomere Shortest Length Assay) (Lai et al., 2017).

Interestingly, Group 2 centenarians had longer average telomere length compared with Group 1 centenarians (3.49 ± 0.35 vs. 2.85 ± 0.24 kb, respectively, *p* = 0.025) (Supporting Information Figure S2). Moreover, Group 2 centenarians were also characterized by a particularly low prevalence of critically short telomeres (length of the shortest 20% telomeres: 1.86 ± 0.21 vs. 1.21 ± 0.14 kb in Group 2 vs. Group 1, respectively, *p* = 0.002) (Supporting Information Figure S2).

Since we observed a dramatic difference in overall health status between Group 2 centenarians (healthier: disease count ≤3; MMSE ≥24; IADL ≥5) and Group 1 centenarians (frail: disease count ≥5; MMSE ≤20; IADL ≤3) (Table 1), we divided the remaining 13 centenarians in our population based on these criteria and obtained four additional healthier centenarians and four additional frail centenarians. Out of the remaining five centenarians, either we did not have enough DNA/RNA to run more experiments (three centenarians) or we did not have sufficient detailed medical records (two centenarians). We performed TeSLA on the additional eight centenarians (four healthier and four more frail) and observed that the four healthier centenarians had significantly longer telomeres compared to the four frail centenarians (average telomere length: 3.08 ± 0.16 vs. 2.59 ± 0.15 kb, *p* = 0.004; Shortest 20% telomeres: 1.57 ± 0.21 vs. 1.18 ± 0.07 kb, *p* = 0.012).

Based on these results, we renamed the original Group 2 together with the additional four healthier centenarians as “high‐performing” centenarians (HP Cent) since they are both healthier (disease count ≤3; MMSE ≥24; IADL ≥5) and have longer telomeres. Accordingly, we renamed the original Group 1 together with the additional four more frail centenarians as “low‐performing” centenarians (LP Cent) since they are both more frail (disease count ≥5; MMSE ≤20; IADL ≤3) and have shorter telomeres.

**Figure 3:**
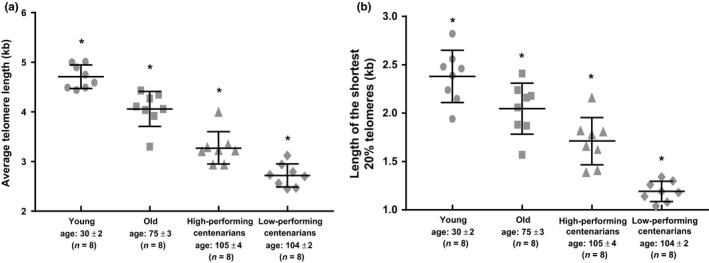
Telomere length measurements by TeSLA (Telomere Shortest Length Assay) in young, old, high‐, and low‐performing centenarians. (a) Average telomere length. (b) Length of the shortest 20% telomeres. *p < 0.05 vs. each of the other groups

We matched the eight HP Cent and eight LP Cent with eight old (75 ± 3 years old) and eight young (30 ± 2 years old). As might be expected, with increasing age, we observed average telomere length shortening as well as a higher prevalence of critically short telomeres (Figure 3a,b). Again, taken together, HP Cent had longer telomeres compared with the cohort of LP Cent (Figure 3a,b). Longer telomeres can be due to inherited genetic factors, differences in life style (e.g., smoking habits, regular exercise and healthy diet), or reduced pathological factors (e.g., less exposure to disease). We found no differences in life style habits between high‐ and low‐performing centenarians (data not shown). However, T cells from HP Cent had a significantly higher telomerase activity upon stimulation (Supporting Information Figure S3a,b), suggesting that longer telomeres in healthy centenarians’ T cells might be associated with a better ability to up‐regulate telomerase following antigen presentation.

### 2.4 Identification of genes potentially involved in healthy aging and healthy T‐cell responses to stimulation

Since HP Cent have relatively long telomeres, an excellent health status based on their age and global gene expression profiles that are more similar to that of young adults, we employed HP Cent as a model to identify genes that might potentially be biomarkers of healthy aging and optimal T‐cell responses to stimulation.

**Figure 4:**
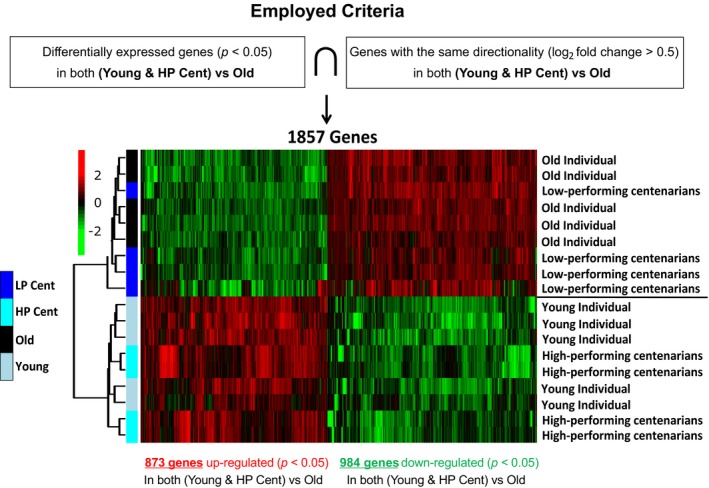
Employed criteria to select genes potentially involved in healthy aging and heat map showing genes differentially expressed between both young and high‐performing centenarians vs. old (67–83 years old) individuals

In order to select potential biomarker genes, we employed a series of stringent criteria (Figure 4). Briefly, we first selected the genes whose expression level was significantly (*p* < 0.05) different between old (67–83 years old) and both young and HP Cent. The expression level of the genes contained in this preliminary list significantly changed in one of the following ways: (a) age‐related increase (young < old < HP Cent); (b) age‐related decrease (young > old > HP Cent); (c) down‐regulation in both young and HP Cent (young and HP Cent < old); (d) up‐regulation in both young and HP Cent (young and HP Cent > old). Genes whose expression changed based on the individual's age (1 and 2 above) are potentially involved in the aging process but may not play a crucial role in promoting healthy aging and extraordinary longevity. We therefore excluded such genes by further selecting only the genes whose expression level was more similar between young and HP Cent compared to the old individuals (3 and 4 above) and with at least 0.5 log_2_ fold difference (Figure 4). While HP Cent are potentially characterized by expression profiles that promote healthy aging and increased lifespan, it is uncertain whether LP Cent also have a predisposition to longevity or they are just “fortunate survivors.” For this reason, we did not employ additional criteria involving the LP Cent.

We finally obtained a list of 1,857 genes whose expression significantly changed in old individuals (67–83 years old) compared with the young subjects but did not change or only mildly changed in HP Cent (Figure 4). Interestingly, 99.4% of these genes (1,846 out of 1,857) had a similar trend in LP Cent as compared to the old (if up‐regulated in both young and HP Cent, they were down‐regulated in both old and LP Cent and vice versa) (Figure 4). Furthermore, 67% of these genes (1,247 out of 1,857) were also differentially expressed (*p* < 0.05) between high‐ vs. low‐performing centenarians (data not shown). This strengthens the hypothesis that HP Cent might potentially have a better ability to regulate the expression of genes involved in healthy aging and “more youthful” T‐cell responses to stimulation.

**Figure 5:**
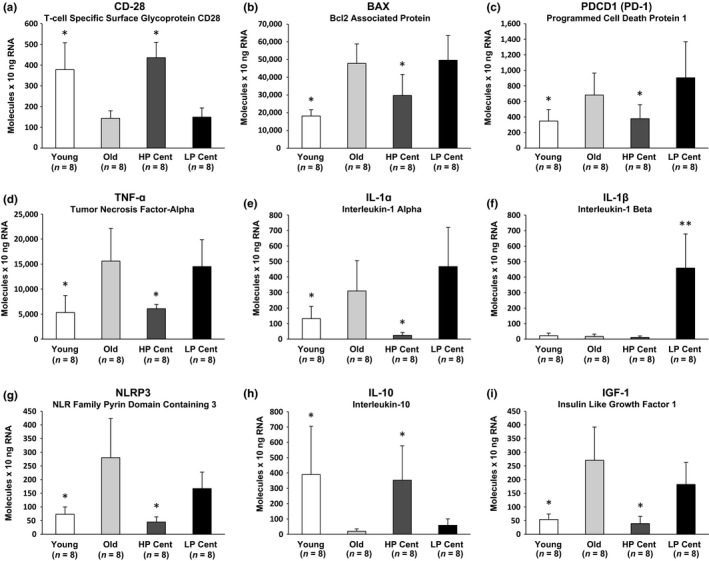
Gene expression levels measured by droplet digital PCR of candidate genes identified by RNA‐sequencing data analysis. Gene expression was measured in T cells after a 72‐hr period of stimulation. A total of eight young, eight old (67–83 years old), eight HP Cent, and eight LP Cent were analyzed. *p < 0.05 vs. both old and low‐performing centenarians; **p < 0.05 vs. all the other groups

We employed this list of 1,857 selected genes to perform a gene set enrichment analysis (GSEA). Genes up‐regulated in stimulated T cells from both young and HP Cent compared to the old were part of pathways involved in processes such as antigen processing and presentation, responses to stimulus, and cellular defense responses (Table 2). This is consistent with the better response to stimulation observed in both young and HP Cent. One important gene within such pathways is the co‐stimulatory receptor CD28, essential for proper T‐cell stimulation. In order to further investigate whether the improved response of T cells from HP Cent is due, at least in part, to an increased expression of the co‐stimulatory receptor CD28, we validated CD28 expression levels using droplet digital PCR (ddPCR). For validation of gene expression, we employed the eight HP Cent, eight LP Cent, eight old (75 ± 3 years old), and eight young (30 ± 2 years old) previously selected by using the criteria illustrated above. Interestingly, stimulated T cells from HP Cent exhibited significantly higher expression of CD28 compared with stimulated T cells from both old individuals and LP Cent (Figure 5a).

**Table 2:** Biological pathways enriched in both young and high‐performing centenarians compared to the old (67–83 years old) by gene set enrichment analysis

Name	NES HP Cent vs. Old	FDR *q*‐value HP Cent vs. Old	NES Young vs. Old	FDR *q*‐value Young vs. Old	Up‐ (↑) or down (↓)‐regulated in both young and HP Cent vs. Old
GO_ACTIVATION_OF_IMMUNE_RESPONSE	2.2888	0.0314	2.2068	0.0219	↑
GO_ACTIVATION_OF_INNATE_IMMUNE_RESPONSE	2.2859	0.0157	2.3343	0.0131	↑
GO_NEGATIVE_REGULATION_OF_B_CELL_PROLIFERATION	2.2643	0.0150	2.3787	0.0144	↑
GO_REGULATION_OF_ACTIVATED_T_CELL_PROLIFERATION	−1.7202	0.0453	−1.9702	0.0334	↓
GO_REGULATION_OF_CELLULAR_COMPONENT_MOVEMENT	−1.9907	0.0490	−2.0423	0.0229	↓
GO_TERPENOID_METABOLIC_PROCESS	−2.0182	0.0411	−2.3142	0.0016	↓
GO_EXTRACELLULAR_STRUCTURE_ORGANIZATION	−2.2221	0.0042	−2.4383	0.0010	↓
GO_REGULATION_OF_EXTRINSIC_APOPTOTIC_SIGNALING_PATHWAY	−2.0373	0.0376	−2.0192	0.0266	↓
GO_NEGATIVE_REGULATION_OF_EXTRINSIC_APOPTOTIC_SIGNALING_PATHWAY	−2.0931	0.0243	−1.8600	0.0426	↓
GO_REGULATION_OF_ANATOMICAL_STRUCTURE_MORPHOGENESIS	−2.0164	0.0385	−1.9537	0.0389	↓
GO_TISSUE_DEVELOPMENT	−2.0301	0.0378	−2.1060	0.0124	↓
GO_NEGATIVE_REGULATION_OF_CELL_DEVELOPMENT	−2.0671	0.0262	−2.2090	0.0047	↓
GO_MULTICELLULAR_ORGANISM_METABOLIC_PROCESS	−2.2696	0.0027	−2.3433	0.0011	↓
GO_MULTICELLULAR_ORGANISMAL_MACROMOLECULE_METABOLIC_PROCESS	−2.3252	0.0010	−2.4407	0.0021	↓

Gene set enrichment analysis of genes down‐regulated in stimulated T cells from both young and HP Cent compared to the old revealed enrichment of genes involved in cytokine‐mediated signaling pathways, apoptosis, and inhibition of cell development (Table 2). Gene set enrichment analysis on the 2,179 genes differentially expressed between high‐ and low‐performing centenarians (Figure 2e) revealed most of these pathways were also enriched (Supporting Information Table S1). Interestingly, both young and HP Cent exhibited down‐regulation of pathways regulating apoptotic signaling (Table 2, Supporting Information Table S1). These pathways are particularly important since they contain apoptotic (e.g., BAX and PD‐1) and inflammatory genes (e.g., TNF‐ɑ, IL‐1ɑ, IL‐1β, and NLRP3) that have been previously reported as potential major drivers of aging and age‐related diseases (Franceschi & Campisi, 2014; Ozaki, Campbell, & Doyle, 2015). We validated the expression levels of these genes by ddPCR.

Consistent with our RNA‐sequencing data, young and HP Cent T cells had reduced expression of both BAX, a key protein involved in p53‐mediated apoptosis (Reyna et al., 2017), and PD‐1, a cell death inducer also involved in cancer and autoimmune diseases (Wang, Sun, Wright, Wang, & Gu, 2016) (Figure 5b,c). Also, young and HP Cent had reduced expression levels of pro‐inflammatory cytokines and increased levels of the anti‐inflammatory cytokine IL‐10 (Figure 5d–h), suggesting that a lower grade of inflammation might protect HP Cent from age‐related disease onset and progression.

IGF‐1 is an additional important gene that is part of the pathways regulating extrinsic apoptotic signaling that emerged from the GSEA analysis. IGF‐1 appears to be important for human longevity and previous studies on centenarians showed that longevity was associated with decreased plasma levels of IGF‐1 and preserved insulin sensitivity (Salvioli et al., 2009). Even though IGF‐1 is mainly produced by other tissues, we found that IGF‐1 expression was significantly down‐regulated in stimulated T cells from HP Cent but not from LP Cent and old individuals (Figure 5i).

### 2.5 Genome distribution of genes associated with healthy aging

We next investigated the distribution of the 1,857 genes more similarly expressed in the young and HP Cent cohorts compared with both the old and LP Cent cohorts (Figure 4). We performed a positional gene enrichment (PGE) analysis for high‐resolution identification of over‐represented chromosomal regions. The strength of such an approach relies on a query optimization strategy (that allows one to virtually consider all the possible chromosomal regions for enrichment), and on the multiple testing correction (which discriminates truly enriched regions vs. those that can occur by chance) (De Preter, Barriot, Speleman, Vandesompele, & Moreau, 2008). We obtained a total of 264 significantly (*p* < 0.05) enriched loci potentially associated with healthy aging (Supporting Information Table S2). These loci were not randomly scattered among chromosomes, but mainly located on chromosomes 1, 2, 7, 12, and 17 (Supporting Information Table S2).

We previously identified a group of genes whose expressions were directly regulated by telomere length (telomere position effects over long distances, TPE‐OLD) (Lou et al., 2009; Robin et al., 2014). In these studies, the presence of long telomeres resulted in a telomere “chromosome loop” approaching genes up to 10 Mb away of the telomere end. In cells with short telomeres, these interstitial telomere loops are lost and the same loci became separated as observed by in situ co‐FISH analyses (Robin et al., 2014). Telomere looping may promote epigenetic regulation of gene expression (it generally silences gene expression). TPE‐OLD is therefore a mechanism by which progressive telomere shortening directly leads to changes in gene expression that, in turn, could contribute to aging and disease initiation/progression long before telomeres become short enough to cause critical DNA damage responses and senescence (Robin et al., 2014). Among the 264 significantly enriched loci (PGE analysis), we investigated the distribution of the 63 regions located within 10 Mb of the telomere ends. These 63 regions (containing genes differentially expressed in both HP Cent and young compared with both old and LP Cent) were not randomly scattered but were mostly located on chromosomes 12p, 14q, 17q, 19q, and 19p (Supporting Information Table S3, Figure S4). Interestingly, the exact same chromosome ends were previously reported to be potentially associated with TPE‐OLD (Robin et al., 2014). Furthermore, a recent genomewide linkage analysis on the DNA sequence of 2,118 nonagenarian Caucasian sibling pairs reported the same chromosome arms (except for chromosome 12p) are associated with longevity (Beekman et al., 2013). Since HP Cent had a very low prevalence of critically short telomeres as opposed to LP Cent (Figure 3a,b, Supporting Information Figure S2), TPE‐OLD may potentially be one of the mechanisms underlying the peculiar gene expression profiles of HP Cent.

## 3 DISCUSSION

Even with the current technology and progress in science and medicine, the probability to live beyond 100 years of age is still extremely rare as the prevalence of centenarians in the United States and Europe is ~22 and 17 centenarians per 100,000 inhabitants, respectively (https://www.pewresearch.org/fact-tank/2016/04/21/worlds-centenarian-population-projected-to-grow-eightfold-by-2050/) (Teixeira, Araujo, Jopp, & Ribeiro, 2017). Furthermore, the probability to live healthily past the age of 100 years is even lower as more than 70% of centenarians are affected by age‐related diseases (Andersen, Sebastiani, Dworkis, Feldman, & Perls, 2012). Some exceptionally long‐lived individuals, the so‐called escapers, can, however, live a mostly disease‐free life for over a century (Andersen et al., 2012), indicating that being “lucky” and/or having a healthy lifestyle is not sufficient and more complicated genetic, epigenetic, and biological mechanisms must take place in order to simultaneously promote healthy aging and extreme longevity.

Nonetheless, these mechanisms are still poorly understood. While there have been previous studies examining exceptionally human longevity, such as in centenarians (Beekman et al., 2013; Franceschi & Bonafe, 2003; Sebastiani & Perls, 2012), almost all of these have been performed using resting PBMC and with samples from heterogeneous populations combining both frail/low‐performing and healthy/high‐performing centenarians. Rather than stratifying individuals simply by chronological age, they may more appropriately be grouped by how well they are aging (biological aging) by evaluating medical records and multiple indicators of health such as BMI, blood pressure, blood glucose, hematocrit, cholesterol, and perhaps most important physical and cognitive functions, together with existing candidate biomarkers under more physiological conditions.

Peripheral blood mononuclear cells, and in particular T cells, act as peripheral biomarkers that mirror alterations within a multitude of organs and tissues (Arosio et al., 2014) and therefore are a useful cell model to study human aging. However, these cells require antigen activation in order to trigger proper defense responses, and once activated undergo dramatic changes in both gene expression and proliferation rate. Our goal was to partially recapitulate in vitro active physiological immune responses and compare them between high‐performing centenarians (“escapers”), low‐performing centenarians (“survivors”), and younger study groups.

Here, we describe how centenarians’ immune cells respond to anti‐CD3/anti‐CD28‐induced stimulation and present global gene expression profiles of centenarian‐stimulated immune cells. Currently, there is still debate whether the age‐associated telomere attrition of immune cells is also coordinated by changes in telomerase activity (Lin et al., 2015), or if the capacity for telomerase expression in human lymphocytes is unaltered with age (Son, Murray, Yanovski, Hodes, & Weng, 2000). Our findings show that upon T‐cell stimulation, telomerase activity decreases with age except for HP Cent who have a more robust telomerase activity up‐regulation that also correlated with increased telomere length and proliferation capacity (Figure 1a–d and 3a,b).

Previous centenarian studies (Beekman et al., 2013; Franceschi & Bonafe, 2003; Sebastiani & Perls, 2012) did not indicate variations in absolute numbers of lymphocytes, and in the present study, we found similar results (lymphocytes × 10^3^/mm^3^ in blood from HP cent, LP cent, and old were 1.67 ± 0.57, 1.78 ± 0.49, and 1.65 ± 0.50, respectively, *p* = 0.836), suggesting that the difference in stimulation‐induced responses between high‐ and low‐performing centenarians is likely due to specific immune cell characteristics rather than their absolute number in the blood. However, the distribution of various T‐cell subsets (e.g., CD4^+^/CD28^+^, CD4^+^/CD28^−^, CD8^+^/CD28^+^, CD8^+^/CD28^−^) could be different. CD28 gradual loss is one of the most profound and consistent events contributing to the age‐associated changes in T cells in humans (Fagnoni et al., 1996). Early studies showed that the loss of CD28 was associated with decreased anti‐CD3 stimulation and decreased ability to up‐regulate telomerase after activation (Huang et al., 2017; Valenzuela & Effros, 2002). In addition, the shorter telomeres in CD28^−^ compared to CD28^+^ T cells (Monteiro, Batliwalla, Ostrer, & Gregersen, 1996) may be due to the observation that CD28 signaling is required for optimal telomerase induction (Huang et al., 2017). It is therefore possible that HP Cent might retain more CD28^+^ cells, leading in turn to a more robust response to the anti‐CD3/anti‐CD28‐induced stimulation and a to more “youthful” global gene expression profile.

Gaining a better understanding of immune cell responses associated with human healthy aging and overall healthspan has important translational implications. To further investigate this topic, we first compared in a subset of our study population global gene expression profiles of stimulated T cells. We observed that some “high‐performing” centenarians were more similar to the young adults than to both the old and the other “low‐performing” centenarians in terms of global gene expression profiles (PCA analysis and Volcano plots) (Figure 2a–e). With the aim to identify genes potentially associated with the high‐performing centenarians’ phenotype, we employed stringent criteria and obtained a selected list of differentially expressed genes (Figure 4). Gene ontology analysis of this gene list (and validation by ddPCR of the expression level of selected candidate genes) revealed that both high‐performing centenarians and the young cohort had up‐regulated pathways also involved in the activation of immune responses and down‐regulated pathways also involved in apoptosis and inflammation (Table 2, Supporting Information Table S1, Figure 5). Decreased expression of pro‐apoptotic factors such as BAX and PD‐1 in HP Cent (Figure 5b,c) could promote lower cellular turnover leading, in turn, to decreased telomere shortening and maintenance of the cell replicative potential.

Epidemiological evidence shows that a state of chronic low‐grade inflammation correlates with many aging‐associated phenotypes (e.g., changes in body composition, energy production/utilization, and immune senescence) and represents a highly significant risk factor for both increased morbidity and mortality (Franceschi & Campisi, 2014). In fact, pro‐inflammatory genes as TNF‐ɑ, IL‐1ɑ, IL‐1β, and NLRP3 are associated with a wide range of age‐related disorders, including cardiovascular, neurodegenerative, and metabolic diseases (Anders, 2016; Croft et al., 2012; Ozaki et al., 2015) and the level of soluble TNF‐ɑ R1 was identified as a predictor of 10‐year all‐causes of mortality (Varadhan et al., 2014). It is therefore consistent that the healthiest centenarians in our study population displayed reduced expression of inflammatory cytokines (Figure 5d–g).

Inflammation does not always lead to tissue degeneration since it is also part of normal tissue remodeling. One factor that can discriminate between adaptive and pathogenic inflammation is the relative strength of anti‐inflammatory responses. Anti‐inflammatory responses not only compensate acute inflammation events but can also partially counteract chronic inflammatory processes (Franceschi et al., 2007). Compared to both LP Cent and the 67‐ to 83‐year‐old cohort, HP Cent also had increased expression of IL‐10, one of the most effective anti‐inflammatory cytokines that protects tissue integrity and attenuates disease severity (Ouyang, Rutz, Crellin, Valdez, & Hymowitz, 2011) (Figure 5h). This suggests that in some exceptional individuals, such as HP Cent, chronic inflammation may develop more slowly or may be restricted or balanced by anti‐inflammatory responses that are less prominent in the general population.

When studying complex multifactorial processes such as longevity and healthy aging, it is of great importance to generate gene lists differentially expressed in the sample subgroups under investigation and to delineate chromosomal regions that are significantly enriched in those genes. Our positional gene enrichment analysis revealed several over‐represented chromosome regions (Supporting Information Tables S2 and S3). Interestingly, only when we investigated the distribution of the enriched regions within 10 Mb of the telomere ends did we obtain results that were comparable to previous studies based on large cohorts of long‐lived individuals (Beekman et al., 2013; Brooks‐Wilson, 2013).

We have previously documented that some human genes can be regulated by the length of telomeres over long distances (TPE‐OLD) (Lou et al., 2009; Robin et al., 2014). Long telomeres promote the formation of a chromatin loop at a distance of up to several megabases from the chromosome end, influencing gene expression over large distances (Lou et al., 2009; Robin et al., 2014). This novel telomere looping mechanism implies that progressive telomere shortening can influence cell physiology and diseases associated with aging long before telomeres become terminally short and inducing a DNA damage signal. We speculate that a more efficient regulation of telomerase activity in high‐performing centenarians’ T cells may be responsible for improved telomere length maintenance mechanisms, leading, in turn, to a differential expression (through TPE‐OLD) of key genes that affect human physiology, aging, diseases, and lifespan. Alternatively, this could be due to the genetic inheritance of longer telomeres or to reduced turnover of hematopoietic stem cells for a variety of reasons. It is also possible that higher inflammaging processes in the old and LP Cent lead to an increased rate of telomere shortening and, in turn, to TPE‐OLD‐dependent changes in gene expression. Finally, TPE‐OLD in long‐lived humans could be chromosome specific; thus, future correlations about single telomere end lengths and gene expression profiles are necessary to further explore this hypothesis.

The focus of our pilot study was to form the basis for the identification of valid and reliable biomarkers associated with mechanisms that regulate healthy human immune cell aging. Our overarching hypothesis is that there are specific candidate genes whose expression greatly differs in activated T cells from HP Cent when compared to those from LP Cent. After further validation in larger study populations, the biomarkers that we have identified may in the future provide personalized geriatric medicine facilitating early diagnosis, prevention, and treatment of age‐related diseases to promote healthy aging. Future studies on centenarians, in particular longitudinal comparisons of immune cell responses over the transition from healthy to unhealthy centenarians’ phenotype, are required to better define the gene expression “signature” of healthy aging.

## 4 MATERIAL AND METHODS

### 4.1 Study design and participants

We performed a cross‐sectional study with PBMCs and T lymphocytes isolated from PBMCs of 114 Caucasian individuals under an Institutional Review Board (IRB)‐approved protocol (certification number STU 042014‐016). Briefly, we recruited 21 centenarians (100+ years old) and 93 individuals aged 23–83 years.

The participant's age at time of enrollment was defined by birth certificates stated by local registry offices and/or dates of birth as stated on passports or identity documents. A trained multidisciplinary staff collected from the enrolled volunteer's information regarding health status, currently used drugs, clinical history, and lifestyle (smoking habits and alcohol consumption). Moreover, past and current disease history was retrieved by an accurate evaluation of the participants' clinical documentations. We were unable to obtain CMV status on the centenarian cohort.

Subjects affected by cancer, infections or autoimmune diseases, or on immunosuppressive treatment at the time of enrollment were excluded from the study. Venous blood samples were drawn from the participants under fasting conditions at the same time in the morning. The study protocol was approved by the Ethical Committee of Saint’Orsola—Malpighi University Hospital (Bologna, Italy), and written informed consent was obtained from all subjects in accordance with the International Declaration of Helsinki.

### 4.2 Peripheral blood mononuclear cell isolation and stimulation

Peripheral blood mononuclear cells were isolated from peripheral blood by centrifugation with Ficoll‐Paque Plus (GE Healthcare) and were then cryopreserved at −140°C pending analysis. Cells were thawed 24 hr prior to mitogen stimulation and cultured in RPMI+GlutaMAX‐I with 10% fetal bovine serum, 1% penicillin, streptomycin, and amphotericin B. After 24 hr, the cell suspensions were transferred into a new flask to remove the monocytes (that adhered to the flask's plastic). Peripheral blood mononuclear cells were stimulated by adding Dynabeads Human T‐Activator CD3/CD28 (Life Technologies) in a 1:1 ratio. After 72 hr of stimulation, Dynabeads were removed using a magnet and cells were cultured for a total of 10 days after stimulation. The percentage of live cells was determined every day by trypan blue exclusion using a TC20 Automated Cell Counter (Bio‐Rad). The cell density was adjusted daily, and when it exceeded 1.5 × 10^6^/ml, cells were diluted with fresh complete RPMI medium to a density of 1.0 × 10^6^/ml.

### 4.3 Telomerase enzymatic activity assay

Telomerase activity was assessed using the droplet digital TRAP (ddTRAP) assay as previously described (Ludlow et al., 2014). Briefly, cells were cultured and counted and 10^5^ cells from each sample were pelleted and stored at −80°C pending analysis. On the day of the experiment, cell pellets were lysed on ice for 45 min with 40 µl of NP‐40 Buffer (10 mM Tris–HCl, pH 8.0, 1 mM MgCl_2_, 1 mM EDTA, 1% (vol/vol) NP‐ 40, 0.25 mM sodium deoxycholate, 10% (vol/vol) glycerol, 150 mM NaCl, 5 mM β‐mercaptoethanol, 0.1 mM AEBSF (4‐(2‐aminoethyl)benzenesulfonyl fluoride hydrochloride)). Two microliters of cell lysate (5 × 10^3^ cell equivalents) were added to a 50 µl extension reaction containing 1× TRAP reaction buffer (10× concentration: 200 mM Tris–HCl, pH 8.3, 15 mM MgCl2), 0.4 mg/ml BSA, 200 nM TS telomerase extension substrate (HPLC purified, 5′‐AATCCGTCGAGCAGAGTT‐3′), and dNTPs (2.5 mM each), incubated for 45 min at 25°C followed by telomerase inactivation at 95°C for 5 min, and then cooled at 4°C.

The ddPCR reaction contained 2 µl of extension products (200 cell equivalents), 1× EvaGreen ddPCR Supermix v2.0 (Bio‐Rad), 50 nM TS primer, 50 nM ACX primer (5′‐GCGCGGCTTACCCTTACCCTTACCCTAACC‐3′) (Ludlow et al., 2014) and dH_2_O to a 20 µl final volume per sample. Droplets were generated exactly as reported (Ludlow et al., 2014) and transferred to a 96‐well PCR plate (twin‐tec 96‐well plate, Eppendorf, Fisher) then sealed with foil (Thermo Scientific, AB0757). PCR was performed in a T100 thermocycler (Bio‐Rad) with a ramp rate of 2.5°C/s between all steps. Activation of Taq polymerase (95°C for 5 min) was followed by 40 cycles of 95°C for 30 s, 54°C for 30 s, and 72°C for 30 s and then cooled at 12°C.

After PCR, the fluorescence was read on a QX200 droplet reader (Bio‐Rad) as previously described (Ludlow et al., 2014). Telomerase activity values were corrected by subtracting background fluorescence (negative control) and normalized, sample by sample, based on the percentage of live cells in culture when the cell pellets were collected. Telomerase activity was expressed as the number of telomerase‐extended TS molecules per cell equivalent (telomerase products per cell).

### 4.4 Shortest telomere length measurement

Genomic DNA was extracted using the Gentra Puregene DNA extraction kit (Qiagen) according to the manufacturer's instructions. Each DNA sample was quantified on a NanoDrop (Thermo Scientific) for concentration and purity, and integrity of DNA was determined as previously indicated (Lai et al., 2017).

The TeSLA method was performed exactly as described (Lai et al., 2017). Briefly, 50 ng of genomic DNA was added to a final volume of 20 µl of ligation buffer containing 1,000 units of T4 DNA ligase (New England Biolabs), 1× Cut Smart Buffer (New England Biolabs), 1 mM ATP, and 1 nM of TeSLA telorettes (TeSLA Telo 1–6) (Lai et al., 2017) and incubated at 35°C for 16 hr followed by heat inactivation at 65°C for 10 min. After ligation, genomic DNA was digested using a set of restriction enzymes (2 units each of CviAII, *Bfa*I, *Nde*I, and *Mse*I, New England Biolabs) as reported (Lai et al., 2017) and then treated with 1 unit of shrimp alkaline phosphatase (rSAP; New England Biolabs) at 37°C for 60 min in a final volume of 50 µl. This mixture was subsequently heat inactivated at 80°C for 20 min, and 10 µl of sample was added to 10 µl of adapter ligation mix (1 μM AT adapter, 1 μM TA adapter, 1 mM ATP, 1× Cut Smart Buffer, and 2,000 units of T4 DNA Ligase) and incubated at 16°C for 16 hr. After adapter ligation, the sample was heat inactivated at 65°C for 10 min and subsequently diluted to a concentration of 15 pg DNA/µl (1:25 dilution). For each sample analyzed, we performed eight independent PCRs (94°C for 2 min followed by 26 cycles of 94°C for 15 s, 60°C for 30 s, and 72°C for 15 min) using a total of 25 µl mix containing 30 pg DNA, 2.5 units of FailSafe enzyme (Epicenter), 1× FailSafe buffer H (Epicenter), and 250 nM primers (adapter and TeSLA TP) (Lai et al., 2017). PCR products were run on a 0.85% agarose gel (1.5 V/cm for 19 hr) followed by Southern blot analysis to detect amplified telomeres as previously described (Lai et al., 2017). Southern blot images were finally analyzed by using MATLAB‐based software to automatically and accurately detect and size annotate the telomere bands including the percentage of shortest telomeres and average telomere length.

### 4.5 RNA sequencing and data analysis

RNA was extracted using Qiagen's RNeasy plus mini kit following manufacturer's protocol and sequenced at the UT Southwestern Genomics Sequencing Core on Illumina HiSeq 2500, 2 × 100 bp configuration. Trimmomatic version 0.32 (Bolger, Lohse, & Usadel, 2014) was used to remove poor quality reads and adapter sequences, with FASTQC performed before and after trimming. Raw counts were obtained using HTSeq (Anders, Pyl, & Huber, 2015) on BAM files generated by aligning trimmed PE fastq files to UCSC human genome version hg19 using tophat 2.0.12 (Trapnell et al., 2010). Normalized counts, generated using DESeq2 (Love, Huber, & Anders, 2014), were used for initial exploratory analysis. Unsupervised clustering and principal component analysis (performed using R, version 3.4.1) were used to visualize the behavior of samples using all genes in which the normalized count was >0 in all samples. Differential gene expression analysis between young and old, young and LP Cent, young and HP Cent, HP Cent and LP Cent, and old and HP Cent was performed using DESeq2 with Benjamini–Hochberg‐adjusted *p*‐value <0.05 as cutoff for significance. Gene set enrichment analysis was performed using default parameters (Subramanian et al., 2005) on log fold change ranked lists of the 1,857 genes identified by employing the criteria shown in Figure 4, and the DESeq2 list of 2,197 genes differentially expressed between HP and LP Cent. Positional gene enrichment (PGE) was performed on the 1,857 gene list (Figure 4) using the PERL script provided by De Preter et al. (2008). Raw data have been deposited on the Sequence Read Archive (SRA) under accession SRP131755.

### 4.6 Statistical analysis

Statistical analysis was performed using the software GraphPad Prism 7.0. Telomerase activity, telomere length, cell proliferation rate, and gene expression by ddPCR were compared between the study groups by univariate ANOVA, followed by Bonferroni's correction. Correlations were performed by linear regression analysis. *p* < 0.05 was used as the threshold value for statistical significance.

## AUTHOR CONTRIBUTIONS

E.T., E.H., and R.O. conducted all experiments. E.T., J.W.S., and W.E.W. conceived and designed the studies and analyzed the data. D.M. and B.A. recruited volunteers, collected blood samples, and isolated PBMC. T.‐P.L. contributed to the development of TeSLA. K.B performed the RNA‐sequencing data analysis and statistical analysis. A.T.L. contributed to the development of ddTRAP assay. E.T. and J.W.S. wrote and edited the manuscript.
