# Supplementary figures and images for: Telomere length and telomerase activity in T cells are biomarkers of high‐performing centenarians
Source: Aging Cell. 2018 Nov 28;18(1):e12859. doi: 10.1111/acel.12859 (PMC6351827; doi:10.1111/acel.12859)

# Supplemental Figure 1

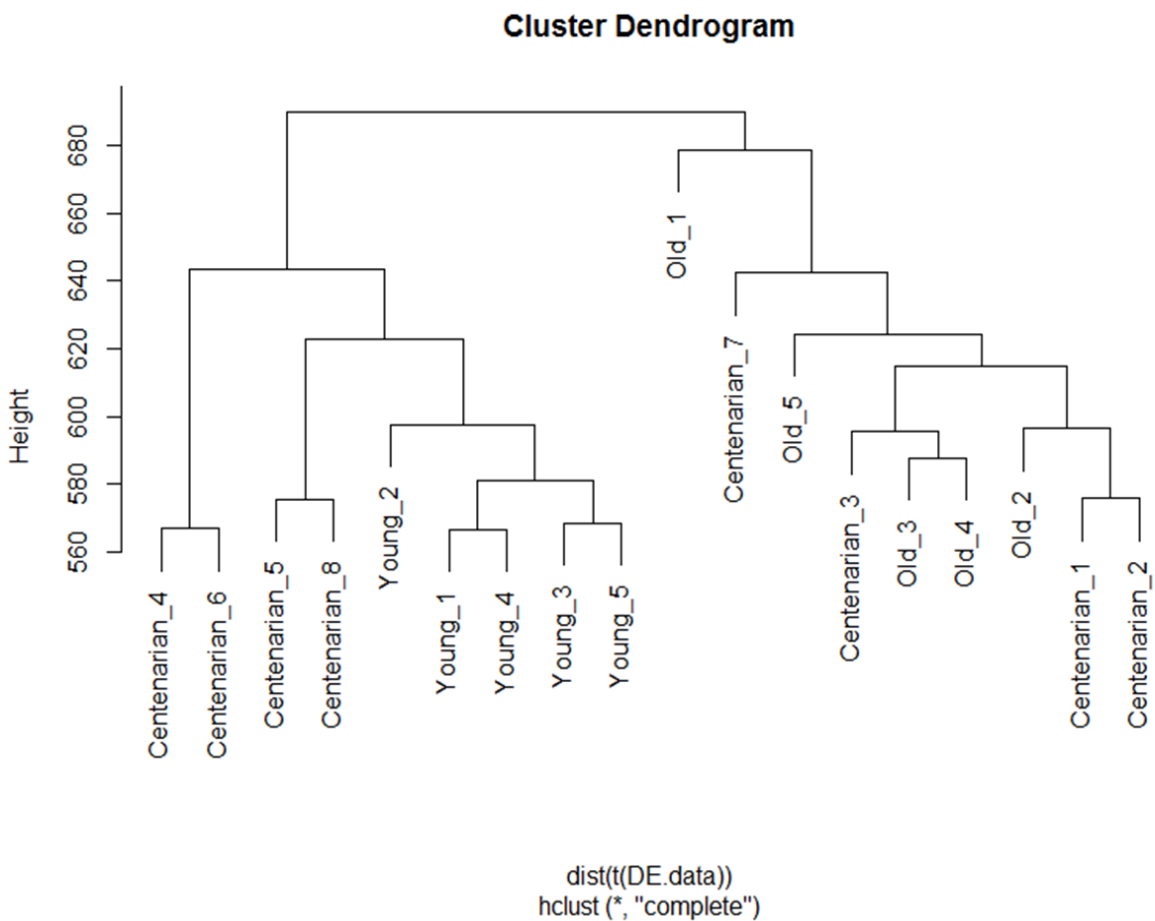

Supplement: Supplementary file 1 [file ACEL-18-e12859-s001.pdf]

Supplemental Figure 2

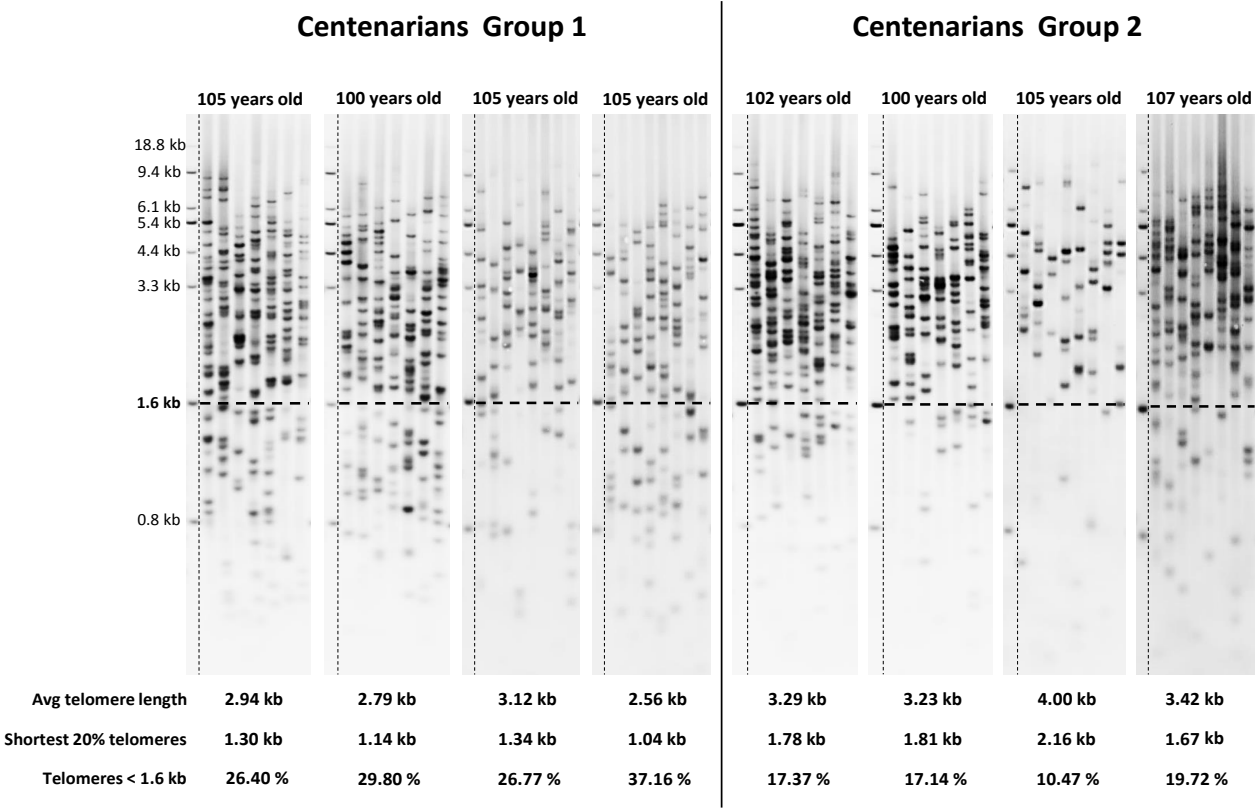

Supplement: Supplementary file 2 [file ACEL-18-e12859-s002.pdf]

# Supplemental Figure 3

a

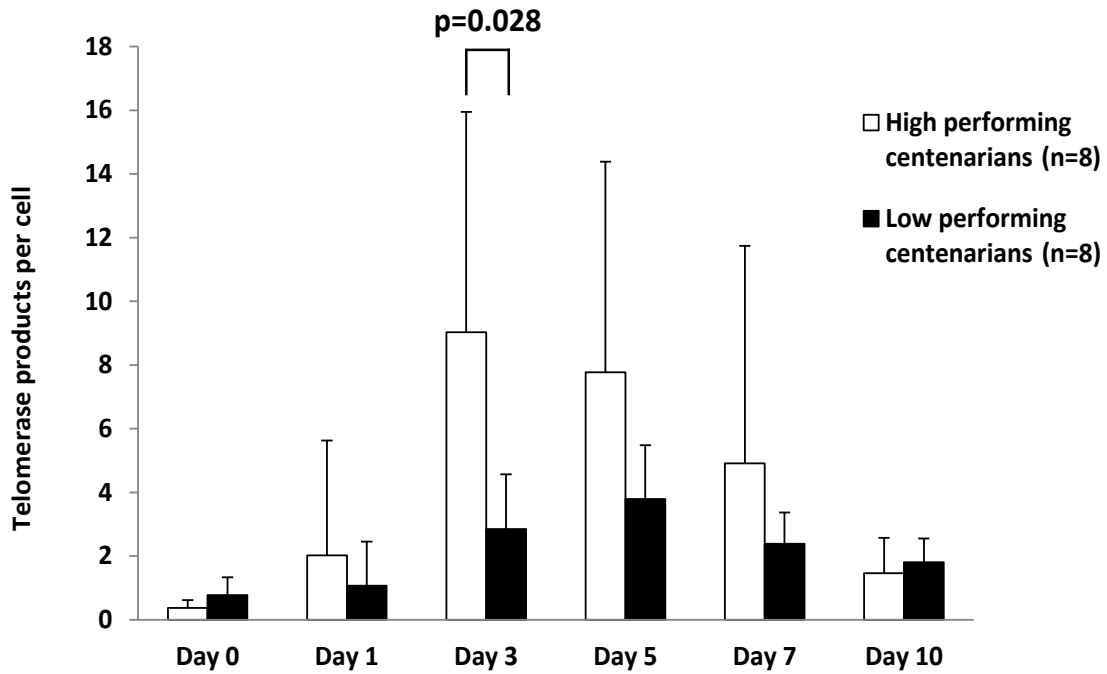

b

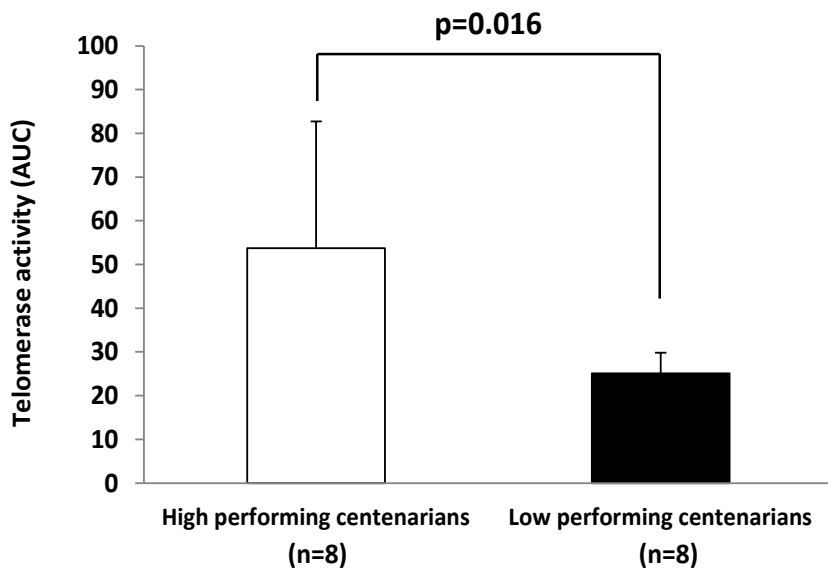

Supplement: Supplementary file 3 [file ACEL-18-e12859-s003.pdf]

# Supplemental Figure 4

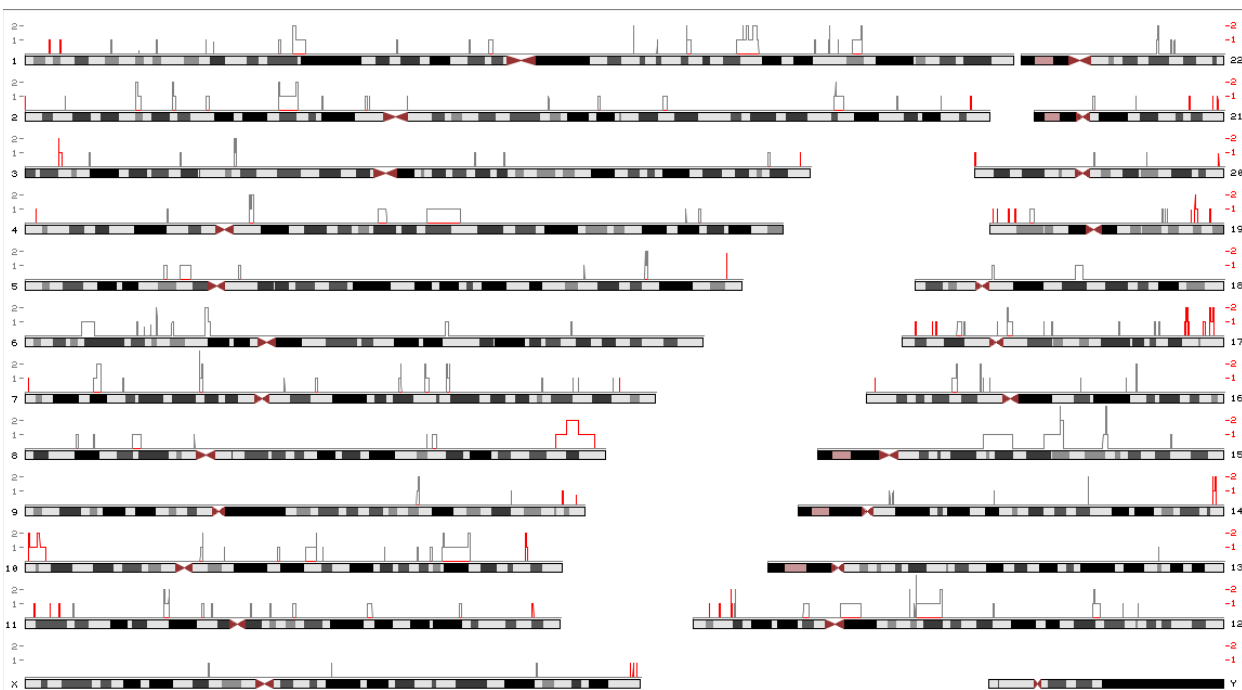

Supplement: Supplementary file 4 [file ACEL-18-e12859-s004.pdf]
