# Supplementary material for: Telomere length and telomerase activity in T cells are biomarkers of high‐performing centenarians
Source: Aging Cell. 2018 Nov 28;18(1):e12859. doi: 10.1111/acel.12859 (PMC6351827; doi:10.1111/acel.12859)
Supplement: Supplementary file 5 [file ACEL-18-e12859-s005.pdf]

Supplemental Table 1

NAME	NES<br>HP Cent vs LP Cent	FDR q-value<br>HP Cent vs LP Cent	Up (↑) or down (↓) regulated<br>In HP Cent vs LP Cent
GO_NEGATIVE_REGULATION_OF_EXTRINSIC_APOPTOTIC_SIGNALING_ PATHWAY	-2.0127	0.0460	↓
GO_REGULATION_OF_ANATOMICAL_STRUCTURE_MORPHOGENESIS	-2.0955	0.0385	↓
GO_TISSUE_DEVELOPMENT	-2.0299	0.0473	↓
GO_NEGATIVE_REGULATION_OF_CELL_DEVELOPMENT	-2.0483	0.0446	↓
GO_MULTICELLULAR_ORGANISM_METABOLIC_PROCESS	-2.1308	0.0330	↓
GO_MULTICELLULAR_ORGANISMAL_MACROMOLECULE_METABOLIC_ PROCESS	-2.1267	0.0283	↓
GO_ANATOMICAL_STRUCTURE_FORMATION_INVOLVED_IN_ MORPHOGENESIS	-2.1670	0.0236	↓
GO_REGULATION_OF_CELL_MORPHOGENESIS	-2.0921	0.0354	↓
GO_BLOOD_VESSEL_MORPHOGENESIS	-2.0830	0.0364	↓
GO_REGULATION_OF_CELL_MORPHOGENESIS_INVOLVED_IN_ DIFFERENTIATION	-2.0633	0.0405	↓
GO_ANGIOGENESIS	-2.1725	0.0315	↓
GO_NEGATIVE_REGULATION_OF_EPITHELIAL_CELL_PROLIFERATION	-2.1775	0.0428	↓
GO_STRIATED_MUSCLE_CELL_DIFFERENTIATION	-2.0184	0.0454	↓
GO_REGULATION_OF_SYNAPSE_STRUCTURE_OR_ACTIVITY	-2.0269	0.0455	↓
GO_REGULATION_OF_EPITHELIAL_CELL_PROLIFERATION	-2.0043	0.0489	↓
GO_WNT_SIGNALING_PATHWAY	-2.0205	0.0467	↓
