# Supplementary material for: Telomere length and telomerase activity in T cells are biomarkers of high‐performing centenarians
Source: Aging Cell. 2018 Nov 28;18(1):e12859. doi: 10.1111/acel.12859 (PMC6351827; doi:10.1111/acel.12859)
Supplement: Supplementary file 6 [file ACEL-18-e12859-s006.pdf]

Supplemental Table 2

PGE analysis of the 1858 selected genes: significantly enriched regions	
# of enriched regions (Total=264)	Chromosome, (# of genes in the enriched regions)
1 to 5	Chr13 (2 genes); Chr18 (8 genes); Chr20 (10 genes); Chr22 (12 genes); Chr21 (11 genes); Chr9 (12 genes)
6 to 10	ChrX (16 genes); Chr14 (26 genes); Chr5 (29 genes); Chr8 (30 genes); Chr4 (38 genes); Chr15 (44 genes)
11 to 15	Chr3 (30 genes); Chr16 (30 genes); Chr6 (34 genes); Chr11 (40 genes); Chr19 (46 genes)
16 to 20	Chr10 (60 genes)
21 to 25	Chr7 (58 genes); Chr2 (65 genes); Chr17 (68 genes); Chr12 (69 genes)
>25	Chr1 (96 genes)
