# Supplementary material for: Telomere length and telomerase activity in T cells are biomarkers of high‐performing centenarians
Source: Aging Cell. 2018 Nov 28;18(1):e12859. doi: 10.1111/acel.12859 (PMC6351827; doi:10.1111/acel.12859)
Supplement: Supplementary file 7 [file ACEL-18-e12859-s007.pdf]

Supplemental Table 3

PGE analysis of the 1858 selected genes: significantly enriched regions between 0-10Mb from the telomere	
# of enriched regions (Total=63)	Chromosome ends, (# of genes in the enriched regions)
0	1q; 4q; 5p; 6p; 6q; 8p; 9p; 12q; 13p; 13q; 14p; 15p; 15q; 16q; 18p; 18q; 21p; 22p; 22q; Xp
1	2p (2 genes); 2q (2 genes); 3q (2 genes); 4p (2 genes); 7p (2 genes); 11q (2 genes); 16p (2 genes); 20q (2 genes); 7q (3 genes); 20p (3 genes)
2	9q (4 genes); 1p (5 genes); 10q (5 genes); 3p (6 genes); 5q (7 genes); 8q (10 genes)
3	21q (6 genes); 11p (7 genes); 17p (7 genes); Xq (9 genes); 10p (12 genes)
4 to 5	12p (12 genes); 14q (13 genes); 19p (16 genes); 19q (16 genes)
>5	17q (28 genes)
